# Investigating the relationship between diffusion kurtosis tensor imaging (DKTI) and histology within the normal human brain

**DOI:** 10.1038/s41598-021-87857-w

**Published:** 2021-04-23

**Authors:** Ahmed Maiter, Frank Riemer, Kieren Allinson, Fulvio Zaccagna, Mireia Crispin-Ortuzar, Marcel Gehrung, Mary A. McLean, Andrew N. Priest, James Grist, Tomasz Matys, Martin J. Graves, Ferdia A. Gallagher

**Affiliations:** 1 Department of Radiology, University of Cambridge School of Clinical Medicine, University of Cambridge, Cambridge Biomedical Campus, Box 218, Cambridge, CB2 0QQ UK; 2 MMIV, Department of Radiology, Haukeland University Hospital, Bergen, Norway; 3 Department of Pathology, Addenbrooke’s Hospital NHS Foundation Trust, Cambridge, UK; 4 Cancer Research UK Cambridge Institute, University of Cambridge, Cambridge, UK; 5 Department of Radiology, Addenbrooke’s Hospital, Cambridge University Hospitals NHS Foundation Trust, Cambridge, UK

**Keywords:** Translational research, Brain

## Abstract

Measurements of water diffusion with MRI have been used as a biomarker of tissue microstructure and heterogeneity. In this study, diffusion kurtosis tensor imaging (DKTI) of the brain was undertaken in 10 healthy volunteers at a clinical field strength of 3 T. Diffusion and kurtosis metrics were measured in regions-of-interest on the resulting maps and compared with quantitative analysis of normal post-mortem tissue histology from separate age-matched donors. White matter regions showed low diffusion (0.60 ± 0.04 × 10^–3^ mm^2^/s) and high kurtosis (1.17 ± 0.06), consistent with a structured heterogeneous environment comprising parallel neuronal fibres. Grey matter showed intermediate diffusion (0.80 ± 0.02 × 10^–3^ mm^2^/s) and kurtosis (0.82 ± 0.05) values. An important finding is that the subcortical regions investigated (thalamus, caudate and putamen) showed similar diffusion and kurtosis properties to white matter. Histological staining of the subcortical nuclei demonstrated that the predominant grey matter was permeated by small white matter bundles, which could account for the similar kurtosis to white matter. Quantitative histological analysis demonstrated higher mean tissue kurtosis and vector standard deviation values for white matter (1.08 and 0.81) compared to the subcortical regions (0.34 and 0.59). Mean diffusion on DKTI was positively correlated with tissue kurtosis (r = 0.82, p < 0.05) and negatively correlated with vector standard deviation (r = -0.69, p < 0.05). This study demonstrates how DKTI can be used to study regional structural variations in the cerebral tissue microenvironment and could be used to probe microstructural changes within diseased tissue in the future.

## Introduction

Imaging biomarkers of tissue microstructure and heterogeneity are clinically important and several have found a role in routine clinical practice, such as the use of water diffusion measurements using magnetic resonance imaging (MRI). In cancer, the non-invasive imaging of such biomarkers has the potential to: characterise tumours and assess grade without the need for a biopsy, enabling earlier monitoring of disease progression and treatment response; and guide clinical management by facilitating patient stratification^1^. These biomarkers also have diagnostic and prognostic use in assessing the structural changes seen in other disease processes, such as ischaemia, demyelination or neurodegeneration^2^.

MRI methods that assess the diffusion of water molecules as a biomarker of tissue microstructure are well-established. Water diffusion results from the random thermal movement of molecules, which can be modelled mathematically as the net movement in a given imaging voxel. In vivo, water diffusion is determined by the structure of the tissue microenvironment, which is dependent on cell number and the contents of the extracellular space^3^. Conventional diffusion-weighted imaging (DWI) can distinguish pathological from normal tissue and has a range of clinical applications. Diffusion tensor imaging (DTI) is an extension of DWI which measures diffusion in multiple directions and has been used to study white matter tracts^4–6^; it allows the measurement of fractional anisotropy (FA), which describes the extent to which diffusion is linearly directional or anisotropic. Despite widespread use of DWI and DTI, these methods rely upon the assumption that molecular water diffusion follows a Gaussian distribution profile of molecular displacements along any given direction. However, biological tissues are complex, structured and heterogeneous environments that restrict diffusion and usually cannot be described by a single Gaussian distribution^7^. Microstructure diffusion methods attempt to overcome this limitation but make a number of assumptions regarding the underlying geometrical properties of the tissue^8,9^.

Diffusion kurtosis imaging (DKI) and diffusion kurtosis tensor imaging (DKTI) are advanced MRI methods that assess the kurtosis of water diffusion^7,10^. Kurtosis, as measured by both DKI and DKTI, is a dimensionless metric that describes the deviation of diffusion from a single Gaussian curve, with large positive values believed to indicate a higher degree of tissue complexity and microstructural heterogeneity^11^. Given that kurtosis may be directional within some tissues, and that DKTI measures kurtosis in multiple directions, this may provide a more accurate characterisation of water diffusion in vivo and could therefore serve as a more accurate biomarker of tissue microstructure and heterogeneity. DKTI may be used to generate parametric maps describing the directionality of kurtosis, providing further insights into the microstructure and heterogeneity of tissue environments. DKTI has been studied in cancer, ischaemic stroke, traumatic brain injury and neurodegenerative disease^12^. Diffusion kurtosis has been used to probe tumour grade in glioma and prostate cancer^13–15^ and to predict tumour responses to therapy in ovarian cancer^16^. Water diffusion can also be used to monitor response to therapy, which highlights that microscopic changes in tumour structure can occur before the macroscopic changes in tumour volume detectable by conventional ^1^H-MRI^17^.

DKTI measures a number of metrics which are listed in Table 1. The dimensionless metric FA measured with DTI describes the degree of anisotropy of diffusion, from a value of 0 (completely isotropic diffusion) to 1 (completely anisotropic diffusion), and indicates the degree of linear structure within the tissue under investigation. Kurtosis fractional anisotropy (KFA) is a novel dimensionless metric studied in DKTI and describes the variation in kurtosis with direction, from a value of 0 (completely isotropic kurtosis) to 1 (completely anisotropic kurtosis), providing additional information regarding the directionality of tissue complexity and microstructure^18^.

**Table 1:** Metrics derived from DWI, DTI, DKI and DKTI.

Parameters	Metric	Coefficient	Units	Method(s)
Apparent diffusion	Mean diffusion	D_mean_	× 10^−3^ mm^2^/s	DWI, DTI
	Axial diffusion	D_axial_	× 10^−3^ mm^2^/s	DTI
	Radial diffusion	D_radial_	× 10^−3^ mm^2^/s	DTI
	Fractional anisotropy of diffusion	FA	Unitless	DTI
Apparent kurtosis	Mean kurtosis	K_mean_	Unitless	DKI, DKTI
	Axial kurtosis	K_axial_	Unitless	DKTI
	Radial kurtosis	K_radial_	Unitless	DKTI
	Fractional anisotropy of kurtosis	KFA	Unitless	DKTI

This study undertook a regional analysis of DKTI in the normal human brain of young volunteers. These metrics were correlated with post-mortem histology, derived from two separate age-matched patients who died from non-neurological causes, in order to investigate the relationship between histological microstructure and DKTI metrics. The study has shown the potential of DKTI for probing tissue microstructure and heterogeneity and has implications for the future use of this approach to study pathological changes in microstructure within diverse disease processes.

## Results

### Volunteer recruitment

Ten healthy volunteers (7 men, 3 women, mean age 23 ± 5 years) were recruited to this study for imaging. One male volunteer was excluded from further analysis due to movement artefact during acquisition.

### Segmentation analysis

**Figure 1:**
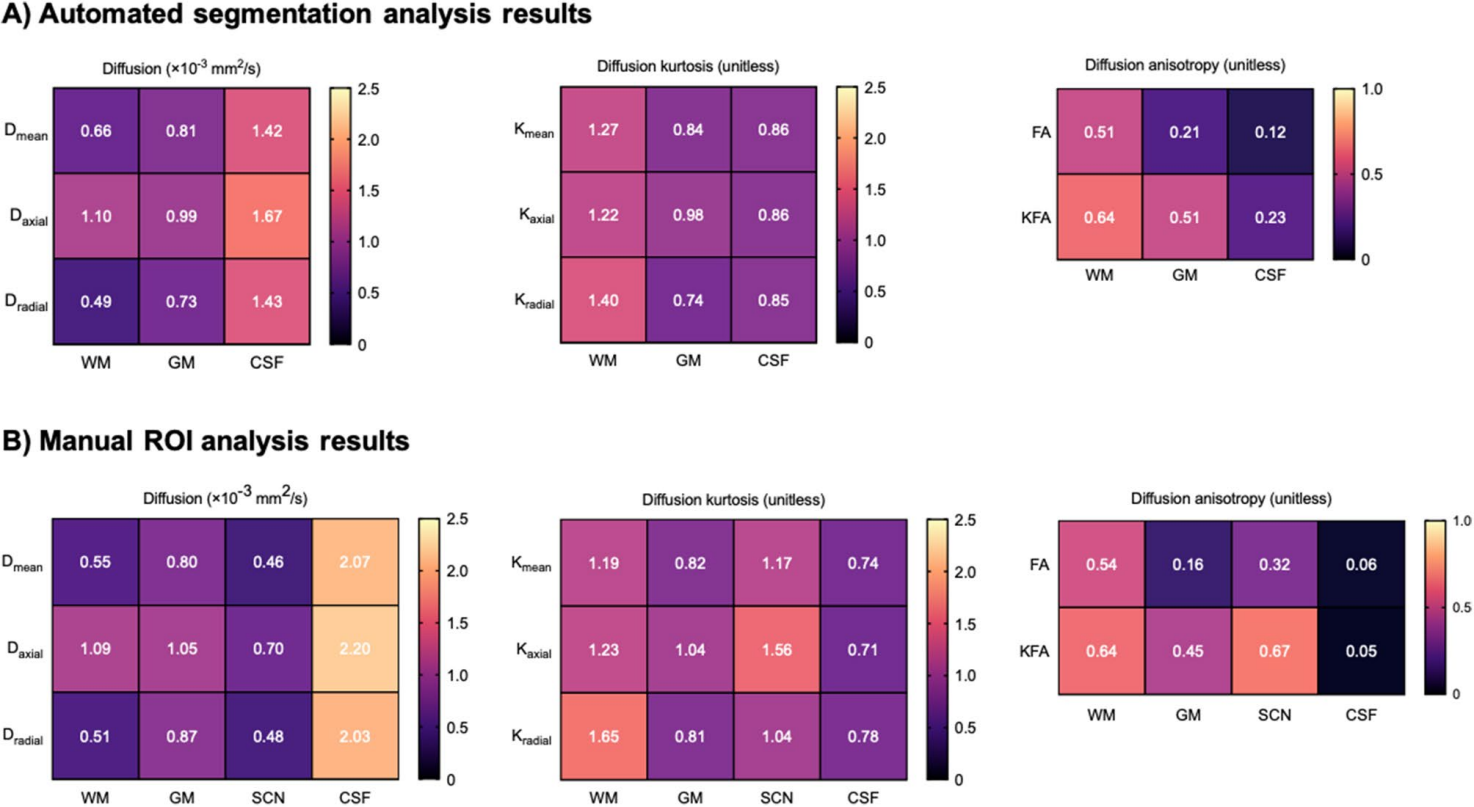
DKTI results for different tissue types. (**A**) Mean DKTI metrics for WM, GM and CSF from automated segmentation analysis. (**B**) Mean DKTI metrics for WM, GM, SCN and CSF derived from all regions in manual ROI analysis.

Segmentation analysis results for WM, GM and CSF are shown in Fig. 1A. Mean, axial and radial diffusion were significantly higher in the CSF (1.42 ± 0.03, 1.67 ± 0.04 and 1.43 ± 0.05 × 10^–3^ mm^2^/s) compared to WM (0.66 ± 0.01, 1.10 ± 0.01 and 0.50 ± 0.01 × 10^–3^ mm^2^/s; *p* < 0.05) and GM (0.81 ± 0.02, 0.99 ± 0.01 and 0.73 ± 0.01 × 10^–3^ mm^2^/s; *p* < 0.05). Mean, axial and radial kurtosis were significantly higher in WM (1.27 ± 0.06, 1.22 ± 0.05 and 1.40 ± 0.06), compared to both GM (0.84 ± 0.03, 0.98 ± 0.04 and 0.74 ± 0.03; *p* < 0.05) and CSF (0.86 ± 0.02, 0.86 ± 0.03 and 0.85 ± 0.02; *p* < 0.05). FA was significantly higher in WM (0.51 ± 0.01) compared to GM (0.21 ± 0.00; *p* < 0.05) and CSF (0.12 ± 0.01; *p* < 0.05), while KFA was also higher in WM (0.64 ± 0.02) compared to GM (0.51 ± 0.02; *p* < 0.05) and CSF (0.23 ± 0.01; *p* < 0.05). Example segmentation maps are shown in Supplementary Figure S1.

### ROI analysis

Figure 1B shows mean ROI analysis results for each tissue type (WM, GM, SCN and CSF). Mean diffusion was significantly higher in CSF (mean 2.07 ± 0.04 × 10^–3^ mm^2^/s) compared to WM (mean 0.55 ± 0.04 × 10^–3^ mm^2^/s), GM (mean 0.80 ± 0.02 × 10^–3^ mm^2^/s) and SCN (mean 0.46 ± 0.07 × 10^–3^ mm^2^/s; *p* < 0.05). Mean kurtosis was similar in WM (mean 1.19 ± 0.06) and SCN (mean 1.17 ± 0.09) regions, which both exhibited significantly higher values compared to GM (mean 0.82 ± 0.05; *p* < 0.05) and CSF (mean 0.74 ± 0.02; *p* < 0.05).

**Figure 2:**
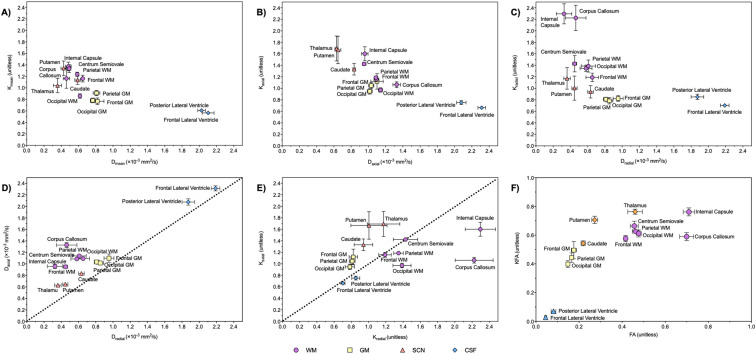
DKTI results for individual ROIs from manual ROI analysis, with comparison between metrics (mean ± SEM). (**A**) Mean diffusion (D_mean_) and kurtosis (K_mean_). (**B**) Axial diffusion (D_axial_) and kurtosis (K_axial_). (**C**) Radial diffusion (D_radial_) and kurtosis (K_radial_). (**D**) Axial diffusion (D_axial_) and radial diffusion (D_radial_). (**E**) Radial kurtosis (K_radial_) and axial kurtosis (K_axial_). (**F**) Fractional anisotropy of diffusion (FA) and kurtosis (KFA). The superimposed dotted lines (*x* = *y*) in (**D**,**E**) separate the diffusion (**D**) or kurtosis (**E**) exhibited by each ROI into predominantly the axial direction (ROI lies above the line) or radial direction (ROI lies below the line).

Figure 2 shows a comparison of ROI analysis results for individual ROIs. Axial and radial kurtosis exhibited a similar pattern to mean diffusion and kurtosis (Fig. 2B,C). High axial kurtosis was exhibited by the internal capsule (WM, 1.60 ± 0.07) and SCN (1.56 ± 0.13) and high radial kurtosis was exhibited by the internal capsule (WM, 2.30 ± 0.12) and corpus callosum (WM, 2.22 ± 0.14). Figure 2D,2E show a comparison of both axial and radial diffusion and kurtosis values for each ROI; the superimposed dotted lines (*x* = *y*) indicate whether the diffusion or kurtosis exhibited by each ROI is predominantly in the axial (above and left of the line) or radial (below and right) direction. Diffusion (Fig. 2D) was predominantly axial for all ROIs investigated and was more prominent in WM regions. In comparison, the CSF spaces exhibited near-equal axial and radial kurtosis values (Fig. 2E) as expected from a freely moving fluid. Kurtosis in the WM had a more diverse profile: frontal lobe WM and the centrum semiovale also showed near-equal axial and radial kurtosis, while the internal capsule and corpus callosum exhibited predominantly radial kurtosis. Conversely, GM and SCN regions demonstrated predominantly axial kurtosis. Mean diffusion and mean kurtosis values for WM, GM, SCN and CSF regions showed low coefficients of variation (Supplementary Table S2).

FA and KFA values (Figs. 1, 2F) were very similar in the CSF (0.05 ± 0.01 and 0.03 ± 0.00) but were different in the WM, GM and SCN (*p* < 0.05). FA values were particularly high in the internal capsule (0.71 ± 0.03) and corpus callosum WM regions (0.70 ± 0.03), and were significantly lower in cortical lobar GM (0.17 ± 0.01) compared to WM (mean 0.62 ± 0.02; *p* < 0.05); the SCN regions of the thalamus (0.46 ± 0.03) and putamen (0.27 ± 0.01) exhibited intermediate values. KFA values were similarly high in the WM (mean 0.68 ± 0.02) and SCN (mean 0.72 ± 0.02) and significantly lower in lobar GM (0.46 ± 0.02, *p* < 0.05). Overall, GM, SCN and lobar WM showed significantly higher KFA compared to FA (*p* < 0.05), while the internal capsule and corpus callosum demonstrated similar KFA and FA values.

### Histological image analysis results

**Figure 3:**
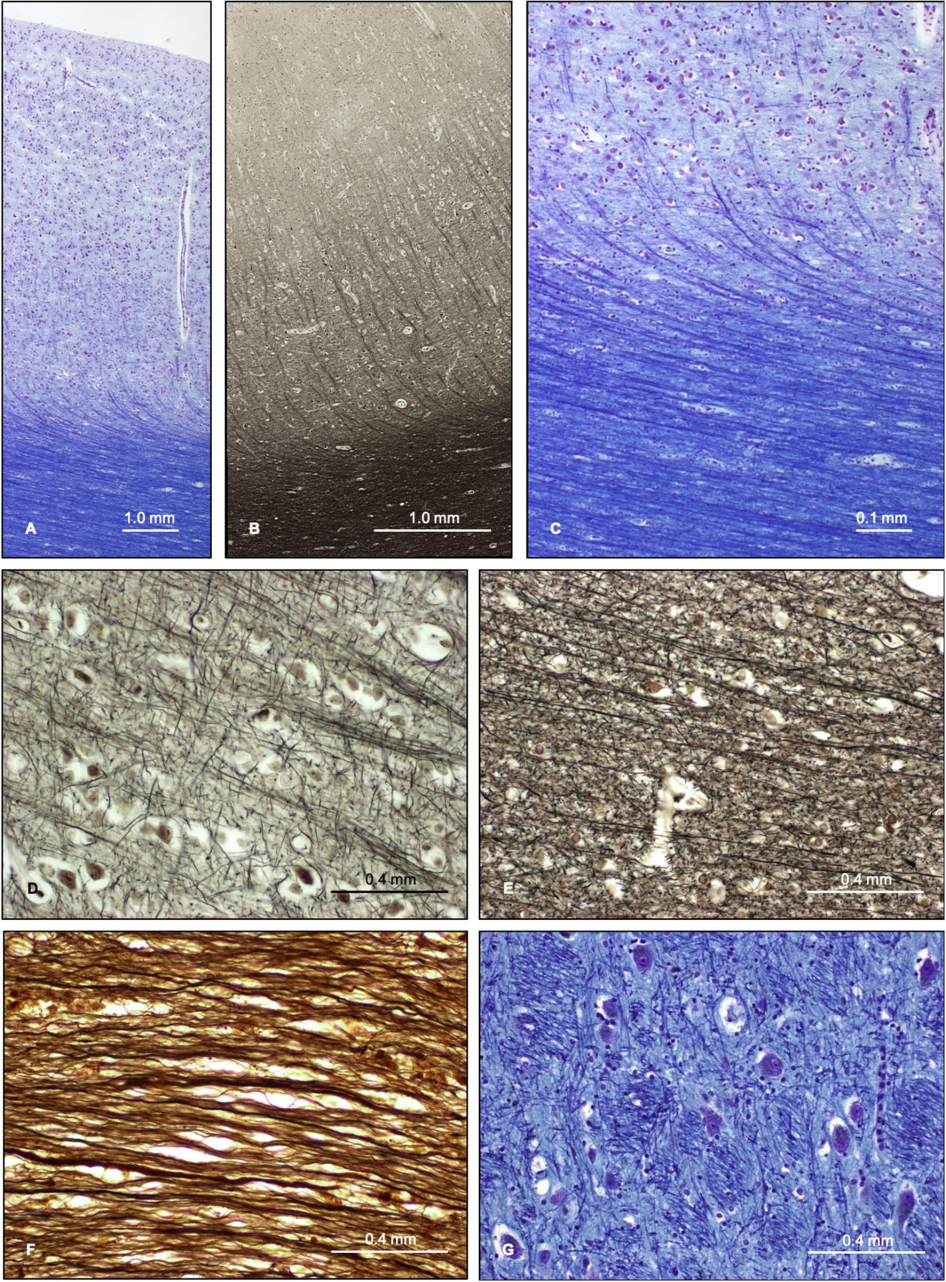
Histology ROI photomicrographs. (**A**–**C**) Full thickness photomicrographs of frontal lobe cerebral cortical GM and underlying subcortical WM, stained with LFB (**A**,**C**) and Palmgren’s silver technique (**B**). (**D**–**F**) Cortical lobe GM (**D**), gyral lobe WM (**E**) and corpus callosum WM (**F**) stained with Glee’s silver method. (**G**) Thalamus stained with LFB.

Qualitative assessment of histological sections of cortical GM, subcortical WM and SCN regions showed features in keeping with previously described work^19^; photomicrographs are shown in Fig. 3. LFB staining for myelin was largely uniform across the lobar subcortical WM and silver staining preparations revealed individual myelinated neuronal fibres with greater resolution than both LFB staining and NFP immunohistochemistry. Staining of lobar cortical GM and subcortical WM demonstrated a sharp delineation between the two tissue types, with a distinct interface between WM and GM. Subcortical WM was shown to comprise myelinated neuronal ‘U fibres’ running in parallel to the cortical surface, before deviating to penetrate the cortical GM. Conversely, the cortical GM exhibited intersecting unmyelinated neuronal fibres orientated in a variety of directions (Fig. 3A–E). Staining of the corpus callosum (Fig. 3F) showed neuronal fibres running predominantly in parallel, following the macroscopic curvature of the structure, but with some regional variations in fibre orientation within smaller fascicles. The deeper WM structure of the centrum semiovale showed a greater variation of fibre orientations. The SCN regions of the striatum (including the caudate and putamen) were shown to comprise GM tissue, containing neuronal cell bodies and unmyelinated neuronal fibres in a variety of orientations, permeated by small bundles of finely myelinated or unmyelinated fibres running in a coherent direction (the ‘pencil fibres of Wilson’); similarly, the thalamus also contained small myelinated WM bundles (Fig. 3G). The densities of cells and neuronal cell bodies for different regions are shown in Supplementary Table S1. WM regions demonstrated high cell densities and conversely, the GM and SCN regions exhibited lower cell densities in the presence of neuronal cell bodies. Regional cell densities were compared with both the mean diffusion and kurtosis values and showed no significant correlation (*p* = 0.50).

**Figure 4:**
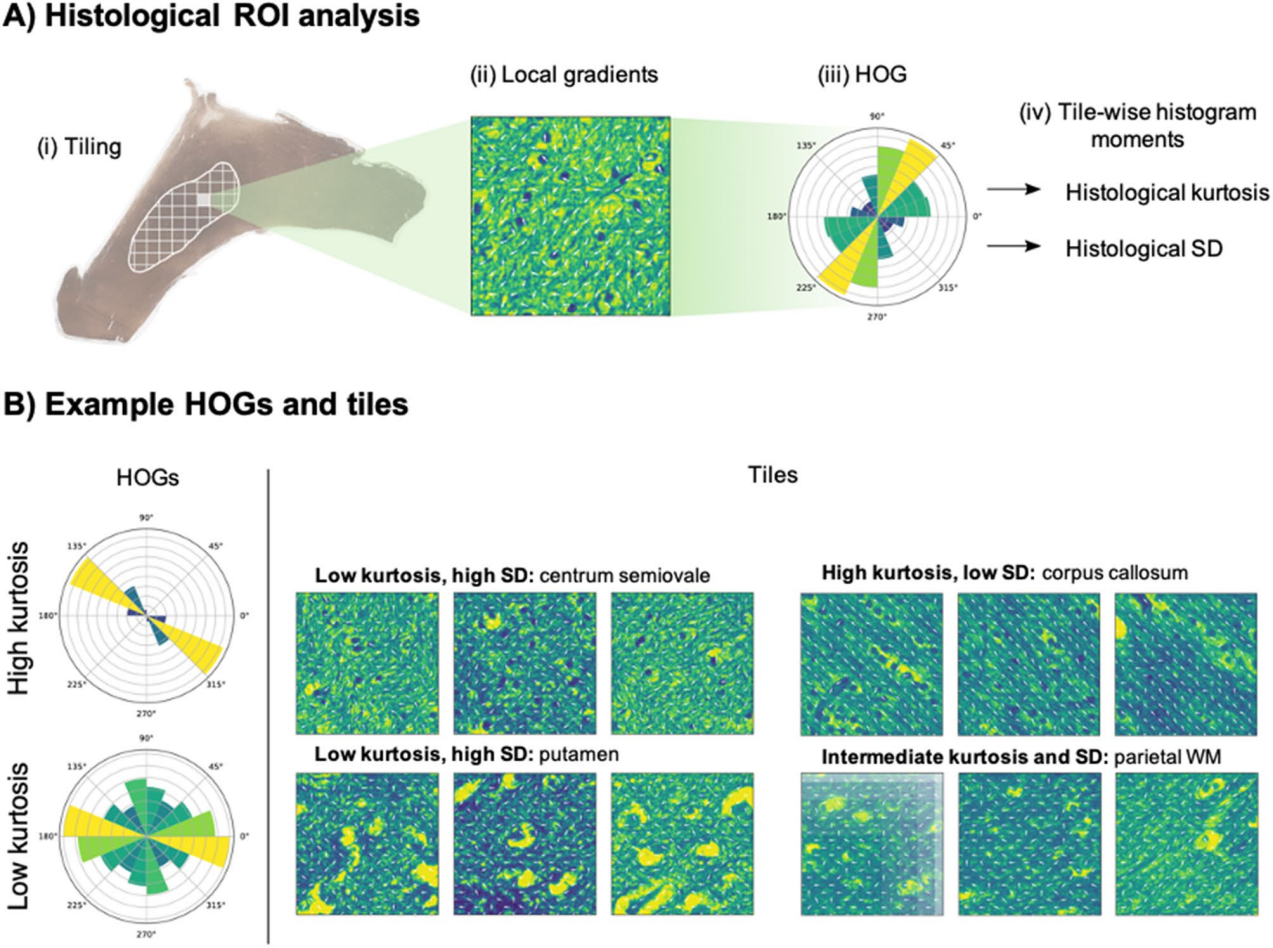
Quantitative histological analysis method. (**A**) Schematic of the histogram of gradients (HOG) generation for histological ROIs. (**B**) Left: examples of HOGs corresponding to tiles with high and low histological kurtosis; right: examples of tiles with different values of histological kurtosis and SD.

**Figure 5:**
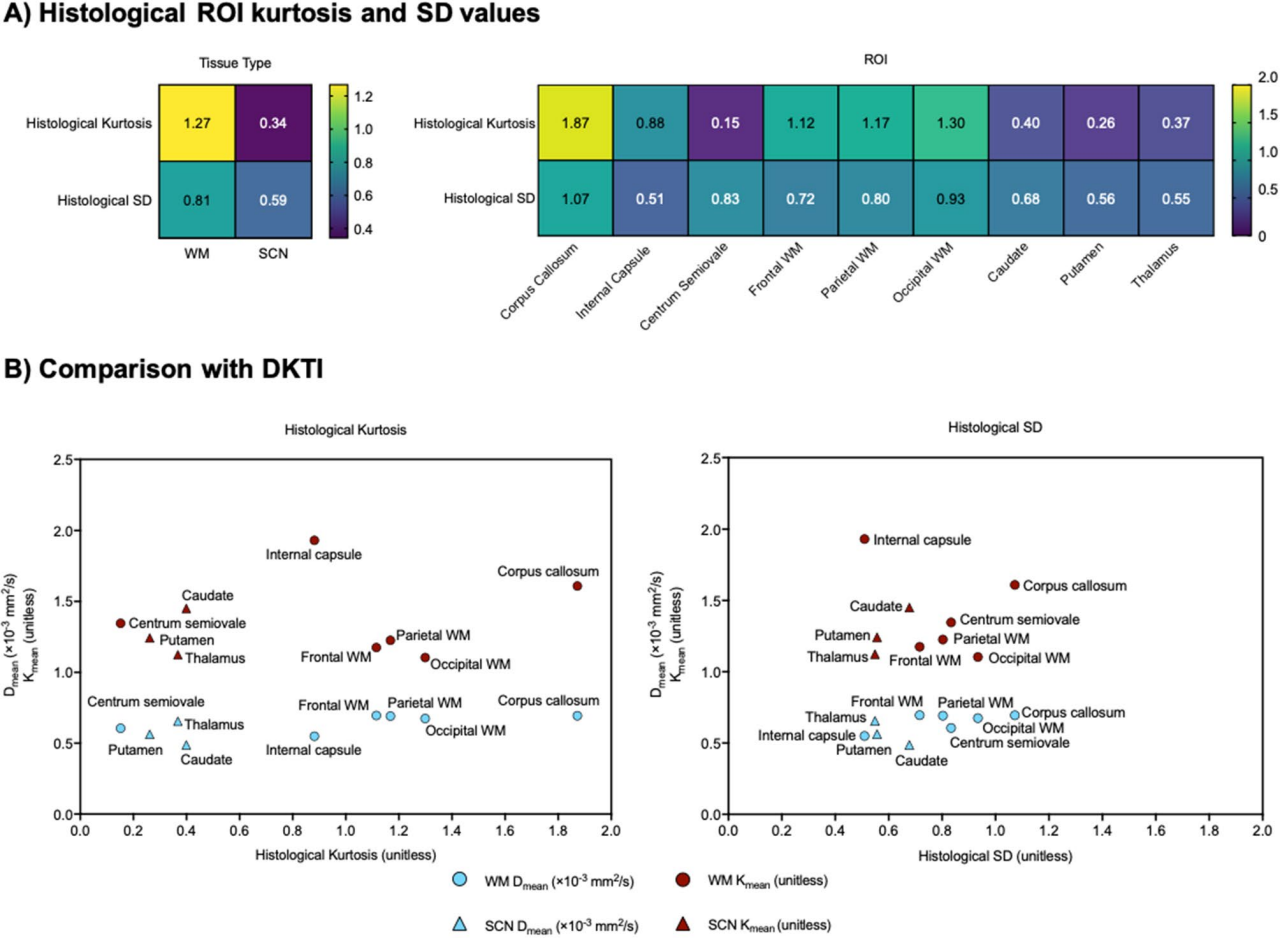
Quantitative histological analysis results. (**A**) Left: mean histological kurtosis and SD values for WM and SCN tissue; right: histological kurtosis and SD values for different WM and SCN ROIs. (**B**) Comparison of histological kurtosis (left) and SD (right) with the DKTI metrics of mean diffusion (D_mean_) and mean kurtosis (K_mean_). D_mean_ was positively correlated with histological kurtosis (r = 0.82, *p* < 0.05) and negatively correlated with histological SD (r = − 0.69, *p* = 0.05).

Analysis of the histological heterogeneity and its directionality was quantified using the feature vector derived from the HOG. Examples of HOGs and corresponding tiles are shown in Fig. 4. The histological kurtosis and SD values for WM and SCN tissue types and individual ROIs are shown in Fig. 5. Overall, mean tissue kurtosis was significantly higher for WM tissue (1.27) than for SCN (0.34, *p* < 0.05). Within the WM regions, the corpus callosum exhibited significantly higher mean histological kurtosis (1.87) than frontal, parietal or occipital lobe WM (mean 1.19, *p* < 0.05). WM regions also demonstrated higher values for the SD of the feature vector (0.81), compared to SCN (0.59); however, the differences in SD values between regions were not found to be significant. These quantitative histological metrics was compared to those from DKTI. Mean and axial diffusion with DKTI were negatively correlated with the SD of the feature vector (r = − 0.69 and − 0.75 respectively; *p* = 0.05 and *p* < 0.05) and positively correlated with tissue kurtosis (r = 0.82 and 0.84 respectively; *p* < 0.05). The remaining DKTI metrics that were investigated (radial diffusion, mean, axial and radial kurtosis, fractional anisotropy of diffusion and fractional anisotropy of kurtosis) showed no significant correlations with tissue kurtosis or tissue SD.

## Discussion

DKTI is an advanced imaging method for studying tissue microstructure and heterogeneity.

This study presents a comprehensive analysis of DKTI metrics within the normal human brain and a comparison to histology across several regions. The results provide some biological explanations for the regional changes in DKTI metrics in different anatomical regions and tissue types within the brain. The brain is an ideal model system to study this imaging technique due to its relatively symmetrical gross structure, low anatomical variation between individuals and well-defined histology, including many directionally orientated structures. In this study we evaluated a homogeneous population of healthy young volunteers to avoid confounding age-related changes in DKTI and tissue microstructure which have been reported previously^20,21^.

Diffusion-based imaging of the brain is used routinely as part of patient care and DKTI could be translated into routine practice following validation as a biomarker for specific clinical indications. Existing studies of DKTI in the human brain have assessed changes in pathology, such as tumours or stroke, but to our knowledge have not compared DKTI with normal histology^22–24^. Other studies have compared DKTI with histology in rodent models^25–27^. In this study, the imaging metrics were compared with histology from age-matched brain donors who died of non-neurological conditions: such post-mortem tissue is difficult to obtain, which accounts for why direct comparison of histological and DKTI metrics in human brain tissue has not been previously performed. This study helps to provide a biological rationale for the use of DKTI as a biomarker of tissue microstructure and heterogeneity.

The WM regions showed low diffusion and high kurtosis, FA, and KFA values: these results are consistent with a structured yet heterogeneous tissue environment. The high FA and KFA values, corresponding to anisotropic diffusion and kurtosis respectively, reflect a high degree of directional organization in keeping with bundles of myelinated neurons travelling in the same direction. In addition, the comparison between axial and radial kurtosis in the WM was consistent with the tissue structure demonstrated on histology. The corpus callosum showed predominantly radial kurtosis, reflecting parallel neuronal fibres crossing between the cerebral hemispheres and was shown to have the highest histological kurtosis. The internal capsule also showed predominantly radial kurtosis, consistent with heterogeneity along the parallel fibres in ascending and descending tracts. The centrum semiovale demonstrated similar kurtosis in the axial and radial directions in keeping with neuronal fibres running in tracts with many different orientations: histologically there is intersection of WM fibres from multiple different tracts including the corona radiata, corpus callosum and superior longitudinal fasciculus. These results suggest that DKTI could be an important tool for assessing the complexity of tract microstructure.

GM showed higher diffusion and lower kurtosis, consistent with a more homogeneous and less directionally organised tissue microenvironment in comparison to WM. This was supported by qualitative histological analysis: photomicrograph staining of cortical GM regions demonstrated a high degree of variation in neuronal fibre orientations, with a sharp demarcation between GM and WM. DKTI analysis indicated that kurtosis in GM regions was more axial than radial, suggesting slightly greater heterogeneity in the anteroposterior direction. GM demonstrated significantly lower FA than KFA, indicating predominantly isotropic diffusion and more anisotropic kurtosis. Interestingly, the subcortical regions indicated a similar pattern to WM, with low diffusion and high kurtosis. This suggests that the SCN examined in this study possess similar diffusion properties to WM, despite being classified histologically as GM tissue. Histological staining of the SCN demonstrated background GM tissue permeated by small WM bundles comprising myelinated neurons running in parallel; the presence of these WM bundles is likely to explain why these regions exhibit features of kurtosis which are more in keeping with WM than cortical GM.

The CSF exhibited high diffusion, low kurtosis, very low FA and KFA, and similar axial and radial kurtosis. These findings are consistent with a homogeneous unstructured environment allowing unrestricted fluid diffusion, as is expected for the contents of the lateral ventricles. In theory, the CSF spaces would be expected to demonstrate a complete lack of kurtosis. The non-zero values seen here could be explained by noise at the high *b *values used, partial volume effects involving periventricular GM tissue during segmentation and ROI analysis, or CSF artefacts from bulk flow or pulsation, which can be common in young people^28^.

Histological analysis included both qualitative appraisal of tissue microstructure and quantitative analysis of neuronal fibre orientation and organisation. Quantitative analyses assessed WM and SCN regions to produce a feature vector derived from a histogram of oriented gradients based on the orientation of neuronal fibres in stained tissue sections and the kurtosis and SD values associated with these as a measure of histological heterogeneity. This approach could not be performed on pure GM regions due to the lack of myelination. WM regions demonstrated higher tissue kurtosis values compared to SCN. The corpus callosum demonstrated the highest tissue kurtosis value, a similar pattern to that seen with DKTI and likely to reflect the complex pattern of neuronal crossing within this structure. The tissue kurtosis of the SCN regions was lower than that for lobar WM, despite similar DKTI metrics, which may be explained by the histological analyses assessing only myelinated neurons, and therefore only accounting for the WM bundles within the SCN without consideration of the significant GM component in these regions. In comparison with the DKTI regional analysis, tissue kurtosis showed a positive correlation with DKTI diffusion and tissue SD showed a negative correlation. This agrees with the expectation that highly organised tissue with low tissue SD and high tissue kurtosis would result in greater displacements of water molecules along the directions of neuronal fibres, reflected in high DKTI diffusion values. No statistically significant correlation was found for other DKTI metrics. Although GM regions could not be assessed using this histological approach, this work represents the first step in validating the biological basis for DKTI and future studies can develop these approaches in larger cohorts.

Conventional measures of diffusion have been shown to correlate with cell density both in vitro and in vivo^29^. This relationship is particularly true in homogeneous normal tissues, in stroke and within many tumour types such as glioma^30–32^. However, the results here show that this relationship is complex within the heterogeneous microstructure of the normal brain, as reflected by the variation and directionality of diffusion kurtosis measurements. These results suggest that diffusion in the normal brain is highly dependent on the complex tissue structure within the microenvironment such as the extracellular composition and fibre orientation rather than tissue cell density alone.

The regional DKTI measurements showed good agreement between individuals in this young cohort, but consequently age-related changes could not be assessed as part of this study. Although the segmentation analysis was automated and highly repeatable, partial volume effects could have contributed to errors in regional measurements. The manual ROI analysis performed in parallel corrected for variations in anatomy and head-positioning between subjects; the operator-dependence of this approach was mitigated by the use of a single operator. Manual ROI analysis yielded similar values to automated segmentation for multiple DKTI metrics and demonstrated low coefficients of variation, indicating good internal consistency using this approach. Automating ROI mask generation based on a manually segmented anatomical atlas may produce more reproducible results in the future. A number of factors could have affected the comparison of the in vivo DKTI and the ex vivo histology metrics. For example, post-mortem tissue, particularly from young patients, is difficult to obtain and thus resulted in a small number of available subjects for comparison. The post-mortem tissue was obtained from individuals who died of non-neurological causes and had no overt abnormalities on qualitative assessment but could have been affected by systemic problems: one of the individuals died from chronic renal failure, which has been associated with white matter abnormalities on DTI, albeit in an older population^33^. Other factors may have affected the comparison of histology and imaging. These include potential individual anatomical variability between the post-mortem histology samples, and processing artefacts when preparing histological sections such as variations in staining intensity between different regions. The discrepancy in spatial scale between the DKTI slices (2 mm) and histological sections (~ 10 μm) is a generic issue with all imaging-tissue comparison studies and the resulting difference in image complexity is a potential source of systematic error. The histological section thickness is predetermined by the need for accurate microscopy and staining, and even if it were possible to combine all consecutive sections together to match the DKTI slice thickness, this would present a significant computational problem given the large amount of high-resolution data that would be involved. In addition, the merged data might not yield discernible image features that could be meaningfully interpreted. The spatial scale discrepancy is partially mitigated by the use of normal tissue, where there is less variation in microstructure over the imaging slice scale than would be expected across a comparable pathological specimen. Similarly, differences in orientation between tissue sections and potential inaccuracies in the co-registration of histological sections and imaging slices may have affected the comparison.

Furthermore, the quantitative histological analysis approach used here only assessed myelinated neurons, which are not the sole determinants of tissue microstructure and heterogeneity as glial cells and the extracellular matrix are also factors in determining the structural properties of brain tissue. Finally, as both the diffusional kurtosis derived from DKTI and histological kurtosis are unitless, a direct numerical comparison between these metrics could not be performed and instead, only respective trends between different tissue types were assessed for correlation.

In conclusion, the results have confirmed that DKTI can be used to map regional differences within the normal human brain, which correspond to histologically distinct tissues. This study relates these results with a histological analysis of cell density, tissue heterogeneity and neuronal fibre orientation, demonstrating that cell density alone is insufficient to account for the quantitative differences in diffusion measured within normal brain tissue. DKTI is non-invasive and could be translated into routine clinical imaging. The quantitative metrics derived provide biomarkers of tissue microstructure and have the potential to complement conventional DWI for the study of both normal and diseased tissue.

## Methods

The study was approved by the regional institutional review board (East of England—Cambridgeshire and Hertfordshire Research Ethics Committee) and written informed consent was obtained from all subjects. Histological tissue was sourced in accordance with ethical protocols from the Cambridge Brain Bank (CBB), which is supported by the NIHR Cambridge Biomedical Research Centre. The collection of tissue by the CBB was approved by the Research Ethics Committee (ref. 20/EE/02083) and informed consent was sought from the next-of-kin or legally authorised representative. Consent was given for the research use of the tissue and in sharing donor age, gender and cause of death.

### Volunteer scanning

This study was approved by a local ethical review committee. Healthy volunteers ≥ 18 years old were recruited for imaging and were required to provide informed written consent. Imaging was conducted using a clinical 3 T MRI system (MR750, GE Healthcare, Waukesha, WI, USA). A volumetric inversion-recovery prepared T_1_-weighted gradient-echo sequence (resolution 0.94 × 0.94 × 1.00 mm, FOV 240 mm, TE 3.2 ms, TR 8.2 ms, IR 450 ms, FA 12 deg.) was acquired for co-registration, tissue segmentation and region-of-interest (ROI) delineation. DWI images were acquired as follows: 30 directions, four b-values (0, 100, 1000 and 2000s/mm^2^), 16 axial slices centred on the splenium of the corpus callosum, dual-spin echo echo-planar imaging, acquired resolution 1.12 × 1.12 × 2.00 mm, reconstructed resolution 0.86 × 0.86 × 2.00 mm, FOV 220 mm, TE 102 ms, TR 2000 ms.

### DKTI map generation

DWI images were registered to corresponding T_1_-weighted images using SPM12 (The Wellcome Trust Centre for Neuroimaging, London, UK). Quantitative maps of DKTI metrics (Supplementary Fig. S2) were generated using DKE 2.06 (NITRC, New York, NY^10^) with spatial smoothing; FWHM [3.0, 3.0, 3.0 mm] and median filtering). The axial and radial metrics are derived mathematically from the eigenvalues of the diffusion tensor: axial diffusion and kurtosis were defined as parallel to the anteroposterior axis, while radial diffusion and kurtosis are perpendicular to this axis^10^.

### Segmentation and region of interest analysis of DKTI maps

**Figure 6:**
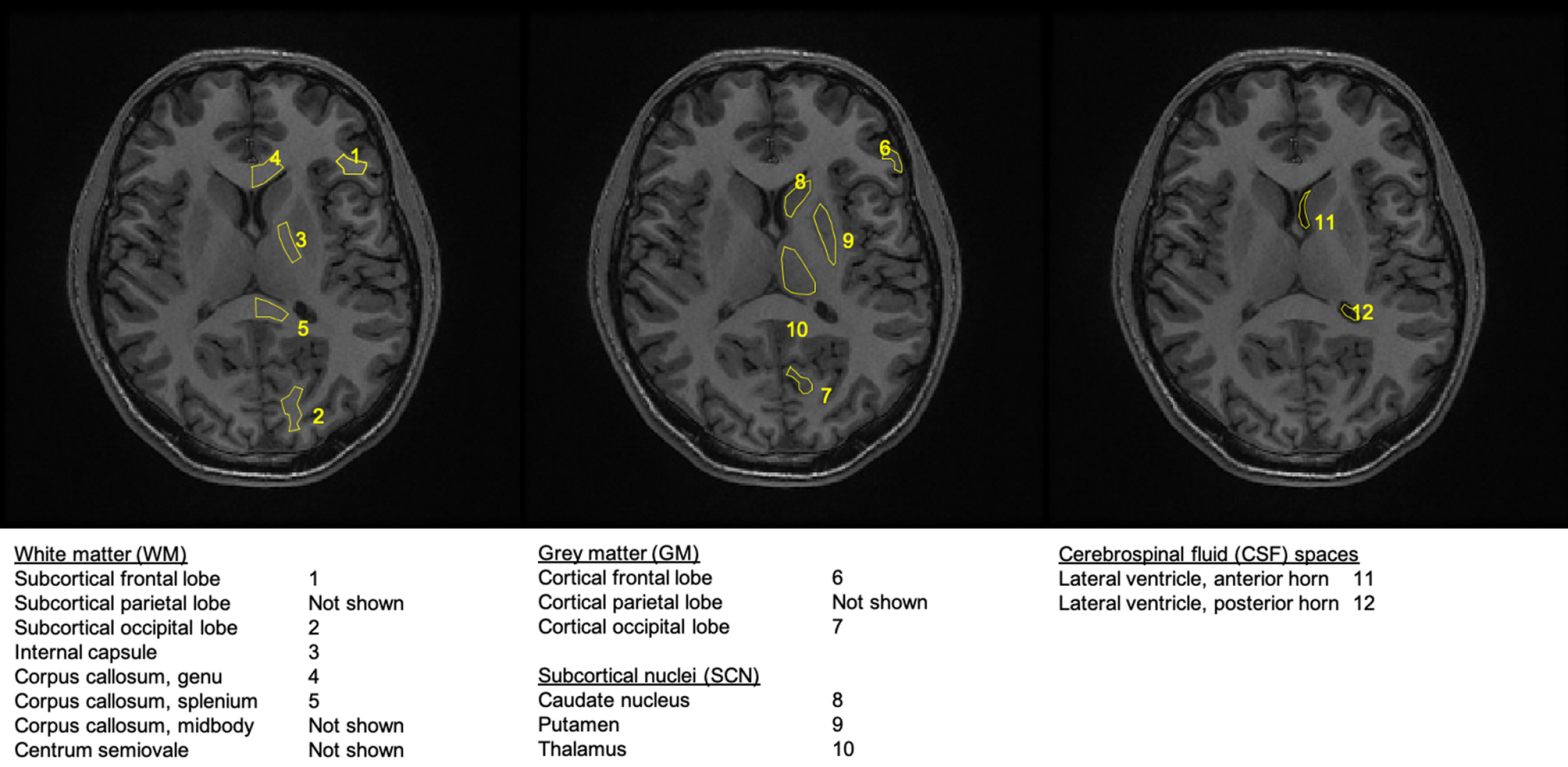
List of all ROIs investigated in the manual DKTI ROI analysis. Corresponding examples are shown on the same T1-weighted image from the same individual. ROIs were selected from WM, GM, SCN and CSF regions and were drawn bilaterally on non-consecutive slices.

T_1_-weighted images were segmented in SPM12 to produce white matter (WM), grey matter (GM) and cerebrospinal fluid (CSF) probability images. These were converted into masks (threshold 95%) which were applied to the co-registered DKTI maps. Mean and standard deviation (SD) values for each metric were calculated for each segmented tissue type. ROIs were selected from WM (cortical and tract), GM (cortical), subcortical nuclei (SCN; caudate, putamen and thalamus) and CSF (ventricular spaces), as shown in Fig. 6. ROI delineation was performed manually to account for variations in anatomy; regions were drawn bilaterally on non-consecutive slices to avoid registration interpolation-related bias and extreme frontal regions were avoided due to image distortions.

### Histology processing and image analysis

Human brain tissue was obtained from two age-matched patients: a male patient who died from chronic renal failure aged 28 years and a female patient who committed suicide by hanging aged 22 years. They both donated brain tissue to the Cambridge Brain Bank. The donor brains were fixed in 10% formalin for 4 weeks before being sectioned in the axial plane at 9 mm intervals. Fixed tissue samples were selected based on the imaging ROIs and were embedded in paraffin, sectioned at 10 µm and mounted on glass slides. To demonstrate brain histology, the slides were deparaffinised and stained with Glees’ silver method and Luxol fast blue (LFB) using standard techniques ^34^. Immunohistochemical staining was used to demonstrate neuronal density (anti-NeuN; Abcam plc, Cambridge, UK) and axonal density (anti-neurofilament protein (NFP; Agilent Dako, Santa Clara, CA, USA). Immunohistochemistry was performed using a Leica Bond Max system (Leica Biosystems, Nussloch, Germany). Sections were pre-treated using heat-mediated antigen retrieval with sodium citrate buffer at pH 6 for 20 min. They were incubated with the primary antibody (anti-NeuN at 5 μg/ml and anti-NFP at 1 μg/ml for 15 min at room temperature) before detection using a horse radish peroxidase (HRP) conjugated polymer system. Diaminobenzene (DAB) was used as the chromogen and the slides were lightly counterstained with haematoxylin. Stained slides were examined with an Eclipse E400 light microscope (Nikon Instruments, Tokyo, Japan) and digitised at 40× using an Aperio AT2 system (Leica Biosystems, USA).

ROIs were drawn on the digitised slides and labelled using QuPath^35^. All the ROIs extracted for histological analysis came from silver-stained WM or SCN regions and were subdivided into non-overlapping 100 × 100 μm tiles and transformed into grayscale using the scikit-image Python library^36^. To quantify and provide a visual presentation of the directionality of the tissue, the histogram of oriented gradients (HOG) was computed for each of the tiles. The process, implemented in scikit-image, included the following steps to ensure robustness: (1) the first-order image gradients were extracted from each tile, which capture the contour, silhouette, and some texture information; (2) tiles were then divided into smaller regions of 20 × 20 pixels termed ‘cells’, and for each of these an 8-bin HOG was determined; (3) to reduce possible effects from variable image brightness, the results were contrast-normalised across local groups of 2 × 2 cells called ‘blocks’, using the L1-norm (sum of absolute values). Finally, the HOG descriptors from each block were combined into a feature vector that describes the entire tile. Figure 4 illustrates this procedure. Standard deviation (SD) and kurtosis values were extracted directly for every feature vector.

### Statistical analysis

Statistical analysis of the DKTI data was performed using GraphPad Prism (GraphPad Software, La Jolla, CA, USA). Two-way ANOVA was performed to compare multiple data groups, with Tukey’s post-hoc test to correct for multiple testing. Comparisons between the DKTI and histology metrics were evaluated using Pearson’s correlation coefficient as implemented in SciPy^34^. *P* values were corrected for multiple testing with the Benjamini–Hochberg method. Significance thresholds were set at 5% (*p* < 0.05).

## Supplementary Information

Supplementary Information 1.

## Abbreviations

ANOVA: Analysis of variance
AP: Anteroposterior
CSF: Cerebrospinal fluid
DKTI: Diffusion kurtosis tensor imaging
DTI: Diffusion tensor imaging
DWI: Diffusion weighted imaging
FA: Fractional anisotropy
FOV: Field of view
GM: Grey matter
KFA: Kurtosis fractional anisotropy
LFB: Luxor fast blue stain
MRI: Magnetic resonance imaging
NFP: Neural fibrillary protein
RBW: Readout bandwidth
ROI: Region of interest
SCN: Subcortical nuclei
TE: Echo time
TR: Repetition time
WM: White matter
