# Supplementary material for: Investigating the relationship between diffusion kurtosis tensor imaging (DKTI) and histology within the normal human brain
Source: Sci Rep. 2021 Apr 23;11:8857. doi: 10.1038/s41598-021-87857-w (PMC8065051; doi:10.1038/s41598-021-87857-w)
Supplement: Supplementary file 1 — Supplementary Information 1. [file 41598_2021_87857_MOESM1_ESM.docx]

**Supplementary Figures**


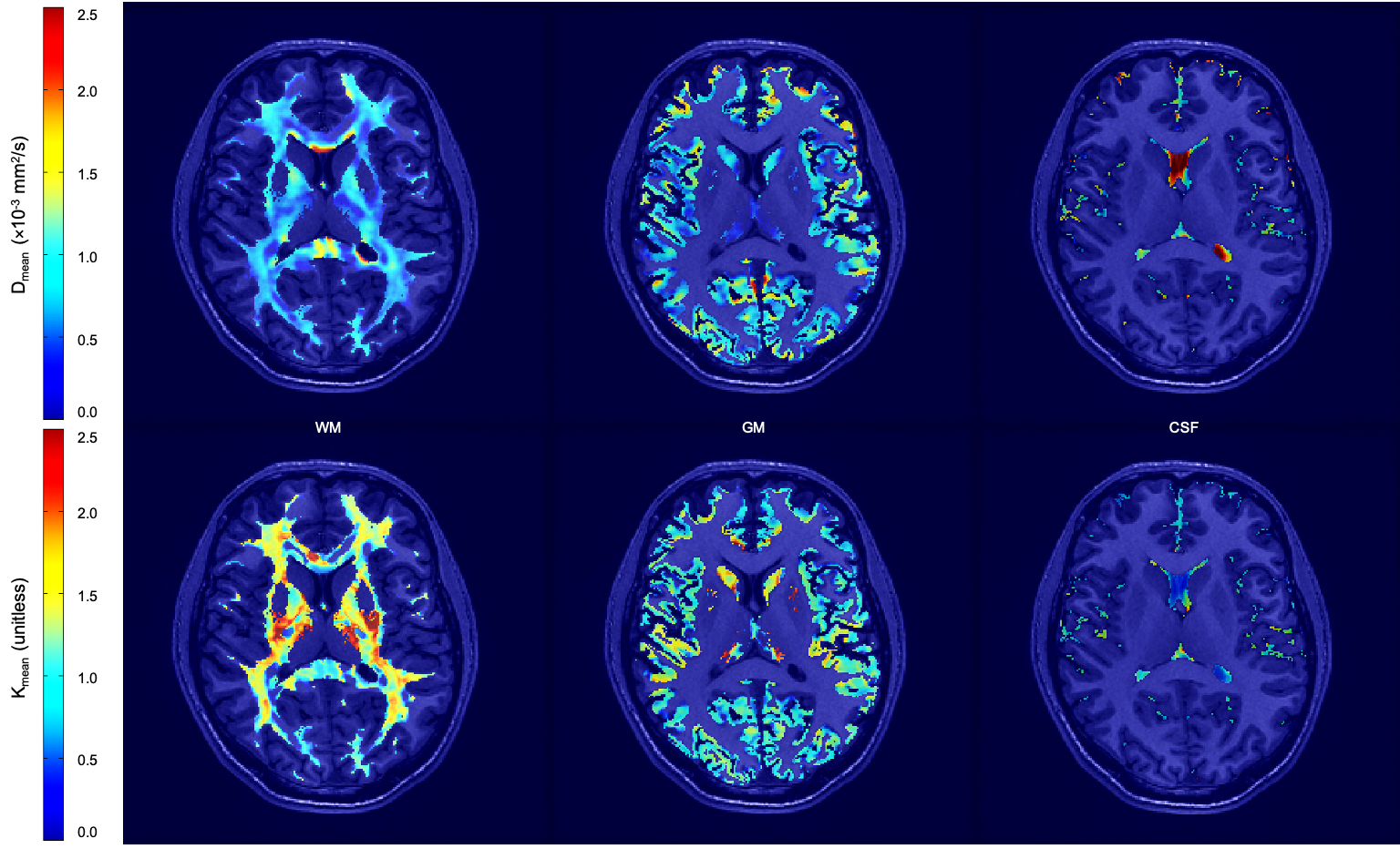
**Supplementary Figure 1**. Example mean diffusion (D_mean_) and mean kurtosis (K_mean_) maps for WM, GM and CSF regions yielded during segmentation analysis. The example maps are derived from the same imaging slice from the same individual and are shown as overlays on the corresponding T1-weighted image.

**
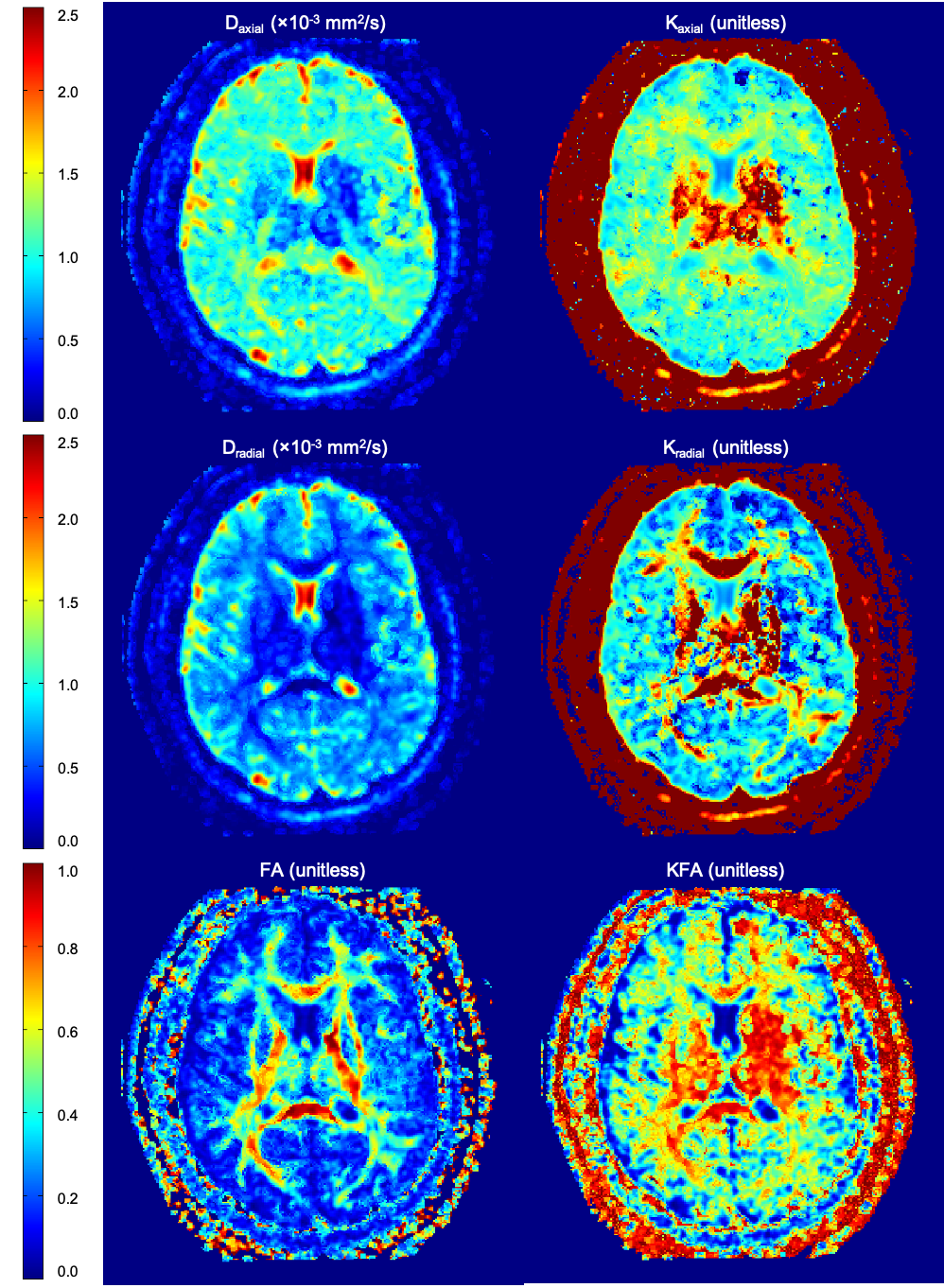
**

**Supplementary Figure 2**. Example DKTI metric maps for axial diffusion (D_axial_), axial kurtosis (K_axial_), radial diffusion (D_radial_), radial kurtosis (K_radial_), fractional anisotropy of diffusion (FA) and fractional anisotropy of kurtosis (KFA). The example maps are derived from the same imaging slice from the same individual.

**Supplementary Tables**


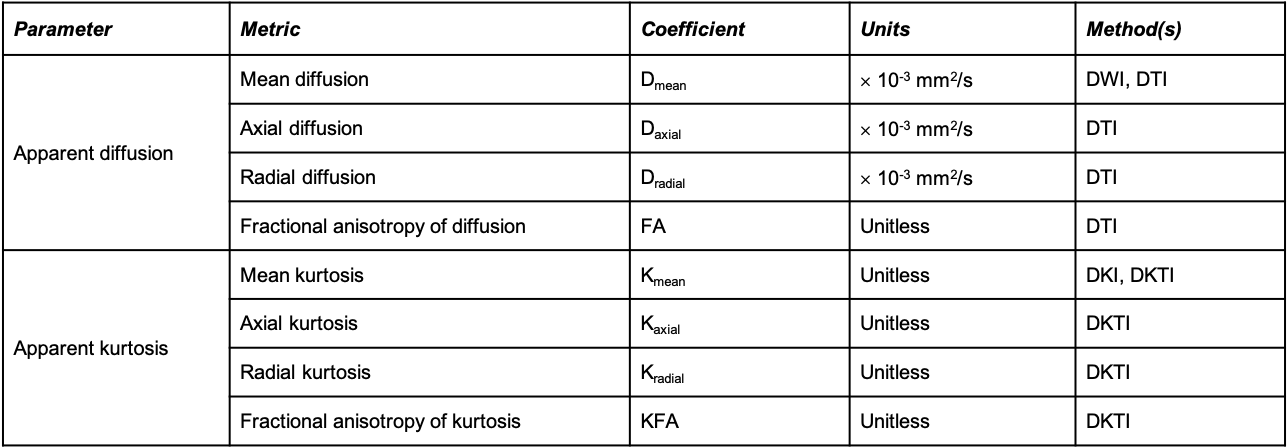

**Supplementary Table 1**. Histology cell counts of total cells and neuronal cell bodies per histological x400 (1.8 µm^2^) field for different ROIs.

**
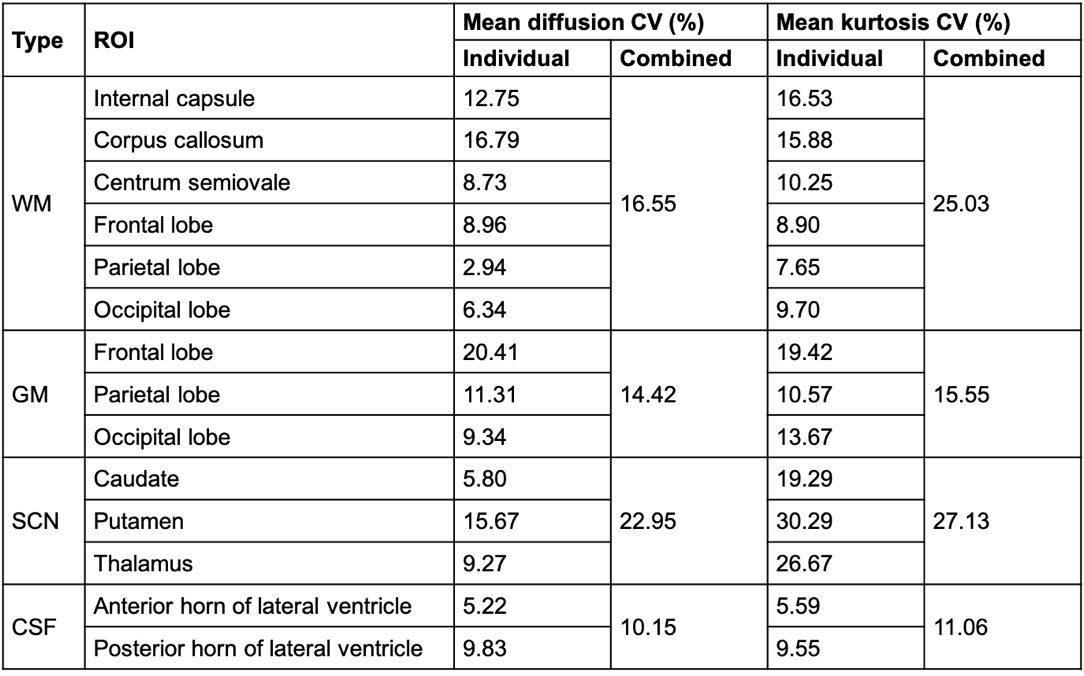
**

**Supplementary Table 2**. Coefficients of variation (CV) for mean diffusion and mean kurtosis for individual ROIs and combined for WM, GM, SCN and CSF tissue types.
